# Relationship between multimorbidity and physical activity: Secondary analysis from the Quebec health survey

**DOI:** 10.1186/1471-2458-8-304

**Published:** 2008-09-05

**Authors:** Catherine Hudon, Hassan Soubhi, Martin Fortin

**Affiliations:** 1 Department of Family Medicine, Sherbrooke University, Quebec, Canada; 2 Health and Social Services Center of Chicoutimi, Quebec, Canada

## Abstract

**Background:**

Abundant literature supports the beneficial effects of physical activity for improving health of people with chronic diseases. The relationship between multimorbidity and physical activity levels, however, has been little evaluated. The purpose of the current exploratory study was to examine the relationship between a) multimorbidity and physical activity levels, and b) long-term limitations on activity, self-rated general health, psychological distress, and physical activity levels for each sex in adults, after age, education, income, and employment factors were controlled for.

**Methods:**

Data from the Quebec Health Survey 1998 were used. The sample included 16,782 adults 18–69 yr of age. Independent variables were multimorbidity, long-term limitations on activity, self-rated general health, and psychological distress. The dependent variable was physical activity levels. Links between the independent and dependent variables were assessed separately for men and women with multinomial regressions while accounting for the survey sampling design and household clustering.

**Results:**

About 46% of the participants were men. Multimorbidity was not associated with physical activity levels for either men or women. Men and women with long-term limitations on activity and with poor-to-average self-rated general health were less likely to be physically active. No relationship between psychological distress and physical activity was found for men. Women with high levels of psychological distress were less likely to be physically active.

**Conclusion:**

Multimorbidity was not associated with physical activity levels in either sex, when age, education, income, and employment factors were controlled for. Long-term limitations on activity and poor-to-average self-rated general health seem related to a reduction in physical activity levels for both sexes, whereas psychological distress was associated with a reduction in physical activity levels only among women. Longitudinal studies using a comorbidity or multimorbidity index to account for severity of the chronic diseases are needed to replicate the results of this exploratory study.

## Background

Finding strategies to deal with chronic medical conditions is a priority for improving the health of the world population [1]. Studies [2-4] in various countries demonstrate that many people have multimorbidity, defined as the co-occurrence of two or more chronic diseases [5]. Persons with multimorbidity require more frequent appointments and hospitalizations [2,6-9] and are at greater risk for drug interactions, acute deteriorations, disability, and mortality [10-14]. They also have higher psychological distress [15] and their quality of life is affected [16,17]. Among the strategies for improving the health of people with chronic diseases, physical activity is extensively supported in the published literature [18-20]. Physical activity improves psychological well-being, and decreases stress, anxiety, and the feelings associated with depression [21,22]. Physical activity also decreases pain during the treatment of painful conditions [23], favors resistance and vigor of people with chronic diseases [18], and decreases their risk of developing functional incapacities [19].

However, sixty percent of the world population and more than 50% of American adults do not attain the minimum of physical activity levels recommended by the American College of Sports Medicine and the Centers for Disease Control and Prevention, namely, 30 minutes of moderate physical activity at least five days a week [24]. Levels of physical activity decrease with age, and women are less physically active than men [2]. Physical activity levels are lower among populations with low income or low education [25,26], and among people with functional incapacities or limited ability to be active [25,27]. Studies also suggest a relationship between employment [28,29], perceived health status or psychological distress, and physical activity levels [25,30-32].

Considering the numerous preventive and curative effects of physical activity, health care professionals should get involved in promoting physical activity among their patients with chronic diseases and multimorbidity [18]. We already know that patients with multimorbidity are more likely to have limited activity levels [33], psychological distress [15], and perception of a lower health status [34]. Kaplan et al. examined predictors of frequent and infrequent self-reported physical activity lasting more than 15 min in community-dwelling people ≥ 65 yr of age. Using logistic regression analyses, they found that the absence of chronic conditions was associated with more frequent physical activity in late life [25]. The relationship between multimorbidity and physical activity levels remains however poorly documented among non geriatric people. A better understanding of this relationship would facilitate development of more appropriate counseling strategies among this clientele.

The purpose of the present exploratory study was to examine the relationship between a) multimorbidity and physical activity levels; and b) long-term limitations on activity, self-rated general health, psychological distress, and physical activity levels for both sexes in adults, after age, education, income, and employment factors have been controlled for. Our hypothesis, suggested by Kaplan's study, was that multimorbidity may be associated with a decreased physical activity level in adults.

## Methods

### Study design and data Source

This study was a secondary analysis of the Quebec Health Survey 1998 (QHS). QHS was approved by the Ethics Committee of Santé Québec. Detailed descriptions of the methods and variables used for the QHS have been described elsewhere [2]. Briefly, the survey was a multistage random probability sample of Quebec residential households designed to collect data about the health and well-being of the Quebec population, and the needs and priority areas for intervention and the allocation of resources. In total, 15,330 households were surveyed. Information about the members of the household was obtained from the person most knowledgeable about the health of family members. The survey, which was in two parts, was conducted in a cascade fashion: one administered by an interviewer; the other, self-administered. The self-administered survey was conditional on having replied to the interviewer survey. The response rate for the interviewer-administered survey was 82.1%. The response rate for the self-administered survey was 69%.

### Participants

We included respondents who were 18–69 yr of age at the time of the survey. Respondents who had cerebral palsy, amputation or long-term limitations on activity after an accident were excluded so that we included only those people with limitations related to chronic health problems. In total, 16,782 persons met these inclusion criteria.

### Independent Variables

The set of independent variables were multimorbidity, long-term limitations on activity, self-rated general health, and psychological distress.*Multimorbidity *(used in the interviewer survey) was conceptualized in two different ways. First, given the exploratory nature of this study, we examined different cut-offs of multimorbidity: starting with 0 as a reference level, followed by 1 chronic condition, then two, three, four and five chronic diseases or more, based on the self-reported diagnosis of conditions from the following list: anemia, dermatologic disease, allergies, back disease, arthritis, serious articular, muscular or tendinous condition, cancer, diabetes, pulmonary condition, mental deficiency, depression, anxiety, psychosis, epilepsy, hypertension, cardiac disease, renal disease, ulcer or other gastric problem, thyroid disease, frequent headache, stroke sequelae, cognitive deficit, obesity, and ocular disease. Second, we also conceptualized multimorbidity as two chronic diseases or more in the same list used by Kaplan: asthma, arthritis or rheumatism, back disease, hypertension, chronic bronchitis or emphysema, diabetes, cardiac disease, stroke sequelae, cognitive deficit and ocular disease. Kaplan's list included also bowel disease but this disease was not evaluated in the survey database. *Long-term limitations on activity *(used in the interviewer survey) was a dichotomous variable that assessed the absence or presence of any self-reported limitation on activity because of a chronic disease or health problem, in comparison with the activity level of people of the same age. *Self-rated general health *(used in the self-administered survey) provided a global assessment of the person's perception of his or her overall health status compared with that of others of the same age. Responses were grouped into two categories: good-to-excellent and poor-to-average. *Psychological distress *(used in the self-administered survey) was measured with a modified version of the Psychiatric Symptom Index of Ilfeld [35]. A high score indicated a greater level of reported psychological distress. The scores were grouped into two categories: low-to-average and high. All independent variables were used as dummy variables with the first category as the reference category. Finally, *socio-demographic characteristics *– age, education, income, and employment – that might account for part of the variance in physical activity levels [2,25,28,29] were included as covariates in the analyses.

### Dependent Variables

*Physical activity level *(used in the self-administered survey) represented the number of leisure physical activity sessions of 20–30 min in the last three months [36]. Responses were grouped into three categories: none, less than three times a week, and more than three times a week.

### Statistical Analyses

All analyses were conducted with SUDAAN software version 9.0 that helped account for the complex survey design and household clustering. Assessment of the associations between the independent variables and the dependent variable was done with a generalized multinomial logit model. Regression parameters were then estimated with the use of generalized estimating equation with a robust variance estimation. The Wald F Test of significance was used with a *P *value set at 0.05.

Data for men and women were analyzed separately. All covariates (age, education, income, and employment) were the first introduced into the generalized logit equations and were maintained as control variables in all models. After the main effects of the independent variables were calculated, the interactions between multimorbidity, long-term limited mobility, self-rated general health, and psychological distress were calculated. Two separate multinomial regressions were run for each of the two forms of conceptualization of multimorbidity (as different levels up to 5 or more chronic conditions, and based on Kaplan et al study's set of diseases). Results were expressed as odds ratios with their *P *values and 95% confidence intervals.

Using a simulation algorithm in SUDAAN software, the power to detect a value ≤ -0.30 (Odds ratios ≤ 0,74) for any parameter for multimorbidity (0, 1, and ≥ 2 diseases) was at least 70.6% for men and 89.2% for women.

## Results

Table 1 presents the characteristics of the study sample: 16,782 persons (46% men) were included in the study. Of these men (n = 7,776), 60% (n = 4,525) were 18–44 yr and 40% (n = 3,251) were 45–69 yr of age. Of the women (n = 9,006), 59% (n = 5,385) were 18 to 44 yr and 41% (n = 3,621) were 45–69 yr of age.

**Table 1:** Characteristics of the study population

	**No. (%)**	
	
**Characteristic**	**Men **(n = 7,776)	**Women **(n = 9,006)
**Age**		
18–44 yr	4525 (60.3)	5385 (59.0)
45–69 yr	3251 (39.7)	3621 (41.0)
**Relative education**		
Lowest	1808 (19.8)	1852 (20.9)
Low	1565 (18.7)	2006 (22.6)
Average	1371 (17.8)	1868 (21.1)
High	1666 (22.8)	1531 (17.3)
Highest	1198 (20.9)	1602 (18,1)
**Income in Canadian dollars**		
< 6000	809 (11.2)	2397 (28.9)
6000–19,999	1797 (24.8)	2829 (34.2)
20000–39,999	2543 (35.0)	2180 (26.3)
≥ 40,000	2106 (29.0)	874 (10.6)
**Employment**		
Part- or fulltime	6160 (79.3)	5431 (60.3)
Not working	1612 (20.7)	3574 (39.7)
**Number of chronic diseases**		
0	3962 (51.0)	3183 (35.3)
1	1975 (25.4)	2319 (25.7)
2	890 (11.4)	1436 (15.9)
3	443 (5.7)	874 (9.7)
4	247 (3.2)	518 (5.8)
≥ 5	259 (3.3)	676 (7.5)
**Long-term limitations on activity**		
Not limited	7139 (91.8)	8046 (89.4)
Limited	635 (8.2)	958 (10.6)
**Self-rated general health**		
Good to excellent	6580 (89.9)	7731 (89.5)
Poor to average	743 (10.1)	905 (10.5)
**Psychological distress**		
Low to average	6290 (83.8)	6724 (77.4)
High	1218 (16.2)	1960 (22.6)
**Physical activity**		
None	2280 (30.0)	2310 (26.2)
< 3 times a week	3422 (45.1)	4329 (49.0)
≥ 3 times a week	1890 (24.9)	2194 (24.8)

None of the interactions that we tested achieved significance in this study. In the following sections, we present the models without the interaction terms. Multimorbidity was not associated with physical activity levels either for men or women when age, education, income, employment, long-term limitations on activity, self-rated general health, and psychological distress were controlled for, regardless of how multimorbidity was conceptualized (Tables 2, 3, 4 and 5). However, compared with men and women with no physical limitations on activity, men and women with long-term limitations on activity were less likely to be physically active. Men and women who rated their general health as poor to average were less likely to exercise than men and women who rated their general health as good to excellent. Finally, no relation between psychological distress and physical activity was found for men. However, women with high levels of psychological distress were less likely to be physically active than women with low-to-average levels of psychological distress.

**Table 2:** Odds ratios, *P *values, and 95% confidence intervals from multinomial logit model linking different levels of multimorbidity, long-term limitations on activity, self-rated health status, and psychological distress levels to physical activity among men.

	**Odds ratio (95% confidence intervals)**		
	
**Independent variables**	**Physical activity < 3 times per week**	**Physical activity ≥ 3 times per week**	***P *value**
Number of chronic diseases			
0	1.00	1.00	
1	0.96 (0.78–1.18)	1.02 (0.81–1.29)	
2	1.06 (0.79–1.41)	0.93 (0.67–1.29)	
3	0.98 (0.68–1.42)	1.11 (0.74–1.66)	
4	1.11 (0.71–1.73)	0.85 (0.49–1.48)	
≥ 5	0.88 (0.52–1.51)	0.74 (0.41–1.33)	0.9273
Long-term limitations on activity			
Absent	1.0	1.0	
Present	0.61 (0.43–0.87)	0.68 (0.46–1.00)	0.0204
Self-rated general health status			
Good to excellent	1.0	1.0	
Poor to average	0.71 (0.53–0.96)	0.52 (0.36–0.76)	0.0018
Psychological distress level			
Low to average	1.0	1.0	
High	1.20 (0.98–1.48)	0.99 (0.76–1.28)	0.0977

**Table 3:** Odds ratios, *P *values and 95% confidence intervals from multinomial logit model linking different levels of multimorbidity, long-term limitations on activity, self-rated health status, and psychological distress levels to physical activity among women.

	**Odds ratio (95% confidence intervals)**		
**Independent variables**	**Physical activity < 3 times per week**	**Physical activity ≥ 3 times per week**	***P *value**
Number of chronic diseases			
0	1.0	1.0	
1	1.03 (0.84–1.26)	1.06 (0.84–1.35)	
2	1.10 (0.87–1.39)	1.00 (0.75–1.31)	
3	0.98 (0.75–1.30)	0.96 (0.69–1.33)	
4	1.04 (0.75–1.45)	1.17 (0.81–1.68)	
≥ 5	0.94 (0.66–1.33)	0.89 (0.59–1.35)	0.9796
Long-term limitations on activity			
Absent	1.0	1.0	
Present	0.68 (0.50–0.93)	0.72 (0.51–1.01)	0.0424
Self-rated general health status			
Good to excellent	1.0	1.0	
Poor to average	0.66 (0.50–0.87)	0.51 (0.36–0.70)	0.0002
Psychological distress level			
Low to average	1.0	1.0	
High	0.82 (0.68–0.98)	0.73 (0.59–0.90)	0.0105

**Table 4:** Odds ratios, *P *values, and 95% confidence intervals from multinomial logit model linking multimorbidity based on Kaplan et al study's set of diseases, long-term limitations on activity, self-rated health status, and psychological distress levels to physical activity among men.

	**Odds ratio (95% confidence intervals)**		
	
**Independent variables**	**Physical activity < 3 times per week**	**Physical activity ≥ 3 times per week**	***P *value**
Number of chronic diseases			
0	1.0	1.0	
1	1.02 (0.84–1.22)	0.78 (0.54–1.12)	
≥ 2	1.01 (0.81–1.26)	0.88 (0.59–1.31)	0.6851
Long-term limitations on activity			
Absent	1.0	1.0	
Present	0.65 (0.46–0.92)	0.66 (0.44–0.97)	0.0298
Self-rated general health status			
Good to excellent	1.0	1.0	
Poor to average	0.73 (0.54–0.99)	0.51 (0.36–0.74)	0.0014
Psychological distress level			
Low to average	1.0	1.0	
High	1.20 (0.98–1.48)	0.98 (0.76–1.28)	0.0913

**Table 5:** Odds ratios, *P *values and 95% confidence intervals from multinomial logit model linking multimorbidity based on Kaplan et al study's set of diseases, long-term limitations on activity, self-rated health status, and psychological distress levels to physical activity among women.

	**Odds ratio (95% confidence intervals)**		
**Independent variables**	**Physical activity < 3 times per week**	**Physical activity ≥ 3 times per week**	***P *value**
Number of chronic diseases			
0	1.0	1.0	
1	1.05 (0.88–1.26)	1.05 (0.85–1.29)	
≥ 2	0.95 (0.71–1.27)	0.84 (0.59–1.18)	0.7332
Long-term limitations on activity			
Absent	1.0	1.0	
Present	0.68 (0.51–0.92)	0.73 (0.53–1.01)	0.0359
Self-rated general health status			
Good to excellent	1.0	1.0	
Poor to average	0.66 (0.50–0.87)	0.52 (0.37–0.72)	0.0003
Psychological distress level			
Low to average	1.0	1.0	
High	0.81 (0.68–0.97)	0.72 (0.59–0.90)	0.0084

## Discussion

To our knowledge, this is the first study to explore the relationship between multimorbidity and physical activity in this age group. We found that none of the cut-offs of multimorbidity (number of chronic conditions) that we used was associated with physical activity levels when long-term limitations on activity, psychological distress, perceived health status, age, sex, education, income, and employment were controlled for.

Our results differ from those of Kaplan's study [25]. Kaplan et al. used data for 12,611 community-dwelling people ≥ 65 yr of age from the 1996–1997 Canadian National Population Health Survey to examine predictors of frequent and infrequent self-reported physical activity lasting more than 15 min, including many independent and confounding variables (geographic location, psychological distress measured with the Generalized Distress Scale, age, gender, educational level, marital status, perceived social support, chronic medical conditions, physical limitations due to injury, functional limitations, smoking behavior and body mass index). Using logistic regression analyses, they found that the absence of chronic conditions was associated with more frequent physical activity in late life. Our two studies shared many similarities: 1) Population Health Surveys; 2) Larges samples; 3) Comparable lists of chronic conditions; 4) Comparable self-reported questionnaires for physical activity; 5) Logistic regression analysis; 6) Separated analysis for males and females. However, Kaplan et al documented a relationship between the absence of chronic conditions and an increased level of physical activity, a relationship that we could not ascertain in our sample. A few hypotheses may be raised to explain different results. First, the ages of our study populations were different. Kaplan et al. [25] recruited people ≥ 65 yr of age, whereas we deliberately excluded people > 69 yr so that we could evaluate a non-geriatric population. Relationship between multimorbidity and physical activity may be different in these two groups. Second, we don't know if the independent "chronic conditions" was considered continuous or categorical in Kaplan's study. This choice may have impact on results. Finally, they include different variables in their model but did not include self-rated general health that is significant in our models. Addition of this variable may have changed their results.

Neither study used a comorbidity or multimorbidity index that accounted for the severity of any single disease or the involvement of a system. One of our previous studies [15] showed that the count of chronic diseases was not associated with an outcome such as psychological distress, whereas chronic disease was associated with psychological distress when an index that takes severity into account was used.

Even if multimorbidity does not seem to be related to physical activity levels, it is related to other variables such as long-term limitations on activity [33], psychological distress [15], and perception of a lower health status [34] that can influence physical activity. Our findings for these variables are in accordance with those of previous studies that demonstrated a relationship between functional incapacities or long-term limitations on activity [27,37-39], perceived health status, and physical activity level [30,31]. We found a significant relationship between psychological distress and physical activity levels only among women. Other studies [25,26,32] demonstrated a relationship between psychological distress and physical activity, but did not distinguish between the sexes.

### Implications for research and practice

Keeping in mind the exploratory nature of our study, results suggest some clues to clinicians regarding the counseling that they can do among patients with multimorbidity. For example, when addressing barriers to physical activity, it may be more appropriate to go beyond the number of medical chronic conditions to evaluate and address long-term limitations on activity, and perception of a lower health status. Effective strategies could also be different for males and females in presence of psychological distress.

Other studies, ideally longitudinal, and using a validated comorbidity or multimorbidity index that takes the severity of diseases into account should be conducted to confirm the relationship between multimorbidity and physical activity levels. Further evaluation is also needed to examine the relationship between psychological distress and physical activity levels of men and women.

## Limitations of the study

The following limitations of this study should be kept in mind. In our analyses we used the variables available from the QHS. First, despite the good quality of the data from the QHS 1998 survey (it had a large sample, four data collections to take seasonal variations into account, and a good response rate), it is possible that recall bias and social desirability may have influenced the respondents' answers. Second, our measure of multimorbidity was limited to a number of chronic diseases. All of these chronic diseases had equal weight in the analysis without any assessment of their severity. Third, although self-efficacy and physical activity levels are strongly related [40,41], the original QHS survey did not measure the relationship between self-efficacy and physical activity prospectively, so we could not include this variable in our model. We were not able to include other variables such as cholesterol, blood glucose, medication and renal function as they were not available in the population survey. Also, other characteristics such as diet, body mass index and smoking may have been included. However, we wanted this exploratory study to be as focused as possible and decided not to include these as covariables in the analysis. Finally, since the data from this study are cross-sectional, we cannot assign any causality to the relationships that were identified. Despite these limitations, our study remains one of the first to explore the topic.

## Conclusion

Multimorbidity was not associated with physical activity levels for either sex in adults when age, education, income, and employment factors were controlled for. Long-term limitations on activity and poor-to-average self-rated general health status seem to be related to lower physical activity levels for both sexes, whereas psychological distress was associated with lower physical activity levels only among women.

## Competing interests

The authors declare that they have no competing interests.

## Authors' contributions

CH participated in the conception and design of the study, supervised analysis, drafted the manuscript and provided funding. HS participated in the conception and design of the study, helped supervise analysis, drafted the manuscript, and provided funding. MF participated in the design of the study, critically reviewed the manuscript and provided funding. All authors gave their final approval for the manuscript submitted for publication.

## Pre-publication history

The pre-publication history for this paper can be accessed here:


